# Bright Silicon Carbide Single-Photon Emitting Diodes at Low Temperatures: Toward Quantum Photonics Applications

**DOI:** 10.3390/nano11123177

**Published:** 2021-11-24

**Authors:** Igor A. Khramtsov, Dmitry Yu. Fedyanin

**Affiliations:** Laboratory of Nanooptics and Plasmonics, Center for Photonics and 2D Materials, Moscow Institute of Physics and Technology, 141700 Dolgoprudny, Russia; khramtsov@phystech.edu

**Keywords:** color centers, silicon carbide, single-photon source, single-photon emitting diode, single-photon electroluminescence, superinjection in homojunctions

## Abstract

Color centers in silicon carbide have recently emerged as one of the most promising emitters for bright single-photon emitting diodes (SPEDs). It has been shown that, at room temperature, they can emit more than 10^9^ photons per second under electrical excitation. However, the spectral emission properties of color centers in SiC at room temperature are far from ideal. The spectral properties could be significantly improved by decreasing the operating temperature. However, the densities of free charge carriers in SiC rapidly decrease as temperature decreases, which reduces the efficiency of electrical excitation of color centers by many orders of magnitude. Here, we study for the first time the temperature characteristics of SPEDs based on color centers in 4H-SiC. Using a rigorous numerical approach, we demonstrate that although the single-photon electroluminescence rate does rapidly decrease as temperature decreases, it is possible to increase the SPED brightness to 10^7^ photons/s at 100 K using the recently predicted effect of hole superinjection in homojunction p-i-n diodes. This gives the possibility to achieve high brightness and good spectral properties at the same time, which paves the way toward novel quantum photonics applications of electrically driven color centers in silicon carbide.

## 1. Introduction

**Figure 1:**
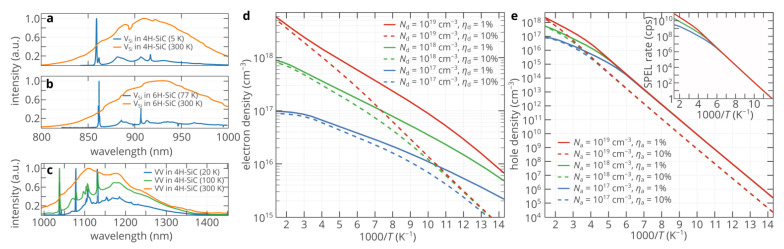
(**a**–**c**) Emission spectra of the V_Si_ center in 4H-SiC (panel **a**), V_Si_ center in 6H-SiC (panel **b**), and divacancy in 4H-SiC (panel **c**) at room and low temperatures. The data are retrieved from [23,24,25]. (**d**,**e**) Temperature dependences of the electron density in n-type 4H-SiC (panel **d**) and hole density in p-type 4H-SiC (panel **e**) for different doping levels and donor and acceptor compensation ratios *η*_d_ and *η*_a_. Inset in panel e shows the temperature dependence of the maximum SPEL rate that can be achieved for the silicon antisite defect at 100% quantum efficiency in p-type 4H-SiC with an acceptor compensation ratio of 1%. The SPEL rate is estimated as a product of the hole capture rate constant [6] and the hole density in p-type diamond.

Color centers in diamond and related wide-bandgap semiconductors are considered one of the most promising emitters for practical single-photon sources (SPSs), which are vital for many quantum information technologies, ranging from unconditionally secure quantum communication lines to optical quantum computers [1,2,3]. For a long time, diamond was considered the leading host material for color centers owing to the exceptionally low electron–phonon interaction, which allows obtaining narrow emission spectra for some point defects, such as the silicon-vacancy (SiV) center, even at room and higher temperatures [4]. However, it recently became apparent that the physical properties of diamond limit the range of its possible practical applications. One of the critical problems is that diamond is more an insulator than a semiconductor. Having a bandgap energy of 5.5 eV, diamond features the activation energy of donors of as high as 0.6 eV [5], which seriously limits the maximum density of free electrons. Therefore, since the single-photon electroluminescence (SPEL) process of color centers is driven by the free electron and hole capture processes [6], achieving bright single-photon electroluminescence (SPEL) of color centers in diamond is extremely challenging [7]. At the same time, electrical excitation of single emitters is essential for many practical applications where scalability, compactness, and energy efficiency are needed since electrical pumping gives the possibility to fabricate hundreds and thousands of micro- or nanoscale SPSs on a single chip and trigger them independently [8]. Silicon carbide recently emerged as a promising alternative to diamond [2,9,10]. As well as diamond, silicon carbide can host optically active point defects, which can be efficiently excited both optically and electrically. The activation energies of donors and acceptors in 4H-SiC are as low as ~0.06 eV [11] and 0.2 eV [12], respectively, which gives the possibility to create high densities of free electrons and holes in the vicinity of the color center and achieve the lifetime limit of the SPEL rate of the color center at room temperature (~100 Mcps) [13,14]. Moreover, the efficiency of electrical pumping can allow the generation of more than 10^9^ photons/s if the lifetime of the excited state of the color center is somehow reduced by more than an order of magnitude (e.g., by using optical nanocavities or nanoantennas [15]) [13]. Thus, at room temperature, single-photon emitting diodes (SPEDs) based on color centers in SiC are superior to any known electrically driven single-photon sources based on other quantum systems, such as quantum dots [2,13,16,17]. The fly in the ointment is that, at room temperature, the emission spectrum of color centers in SiC is not as narrow as that of the SiV center in diamond. The Debye temperature of SiC is approximately twice lower than that of diamond [18], which clearly indicates that the impact of phonons on the emission properties of color centers in SiC is significantly stronger than in diamond (Figure 1a–c). Although a low Debye–Waller factor is not a serious problem for some free-space quantum key distribution protocols and many sensing applications [19,20,21,22], it does not allow, for example, to use room temperature SPEDs based on color centers in quantum optics circuits. The spectral properties of color centers in SiC can be greatly improved by cooling the SiC SPEDs and, therefore, decreasing the electron–phonon interaction (Figure 1a–c). However, as the device temperature decreases, the densities of free charge carriers in the n-type and p-type regions of the SiC SPEDs exponentially decrease (Figure 1d,e). Hence, the ability of these regions to inject free carriers in the active region of the SPED also decreases. Since the SPEL rate is proportional to the density of free carriers in the vicinity of the color center [6], the brightness of SiC SPEDs should also decrease. Figure 1d,e shows that, due to the higher activation energy of acceptors, the density of holes is the limiting factor. Therefore, the maximum SPEL rate at 77 K is expected to be 10 orders of magnitude lower than at room temperature (Figure 1e).

In this work, using a rigorous theoretical approach, we study for the first time the temperature characteristics of the SPED based on single color centers in 4H-SiC. We demonstrate that, although the SPEL rate does decrease as temperature decreases, the rate of this decrease is orders of magnitude lower than what can be expected from Figure 1e, which gives the possibility to achieve both moderately high brightness and good spectral properties at the same time.

## 2. Methods

The theoretical framework used in the article is based on the methodology developed in Refs. [13,14,26]. The SPEL rate and *g*^(2)^ function of the color center are found by solving the system of equations for the populations of the energy levels of the color center [13]. For example, for the silicon antisite defect near stacking faults in 4H-SiC, it can be written as follows [26]:

(1) $$\{\begin{array}{c} \frac{df_{|e\rangle }}{dt}=f_{|+\rangle }c_{n}n-f_{|e\rangle }\left(\frac{1}{\tau_{r}}+\frac{1}{\tau_{\mathrm{nr}}}\right)\\ \frac{df_{|s\rangle }}{dt}=\frac{f_{|e\rangle }}{\tau_{\mathrm{nr}}}-\frac{f_{|s\rangle }}{\tau_{s}}\\ \frac{df_{|g\rangle }}{dt}=\frac{f_{|e\rangle }}{\tau_{r}}+\frac{f_{|s\rangle }}{\tau_{s}}-f_{|g\rangle }c_{p}p\\ \frac{df_{|+\rangle }}{dt}=f_{|g\rangle }c_{p}p-f_{|+\rangle }c_{n}n \end{array}$$

where *n* and *p* are the electron and hole densities in the vicinity of the color center; *c*_n_ and *c*_p_ are the electron and hole capture rate constants, which are determined mainly by the host material rather than the color center itself; *f*_|e>_, *f*_|s>_, *f*_|g>_ are the populations of the excited, shelving, and ground levels of the neutral charge state of the color center, respectively; *f*_|+>_ is the population of the ground level of the positively-charged state; *τ*_r_ and *τ*_nr_ are the radiative and non-radiative lifetimes of the excited state, respectively; and *τ*_s_ is the lifetime of the shelving state. Equation (1) implies that only the neutral and positivelycharged states are involved in the SPEL process. In the case of the color center with negatively charged and neutral charge states, one should swap *c*_n_*n* with *c*_p_*p* and replace *f*_|+>_ with *f*_|−>_. In both cases, a photon is emitted from the neutral charge state. However, we note that the results of the present study do not depend on from which charge state (charged or neutral) a photon is emitted. The SPEL rate under continuous electrical pumping is found as a steady-state solution of Equation (1), while *g*^(2)^(*τ*) = *f*_|e>_(*τ*)/*f*_|e>_^ss^, where *f*_|e>_(*τ*) is the solution of Equation (1) under the initial conditions *f*_|g>_ = 1 and *f*_|e>_^ss^, is the steady-state population of the excited level. The electron and hole densities in the vicinity of the color center used in Equation (1) are found using self-consistent 2D numerical simulations of the electron and hole transport in 4H-SiC SPED based on the Poisson equation, drift–diffusion current equations, and the electron and hole continuity equations performed in Synopsys TCAD. The simulation domain was 70 μm × 17 μm, while the contacts were 30-μm-wide. The mesh size near the p^+^/n^−^ and n^−^/n^+^ junctions and underneath the contacts was as small as 1 nm to correctly simulate the free charge carrier transport. The material parameters used in the numerical simulations are listed in Supplementary Information.

## 3. Results and Discussion

**Figure 2:**
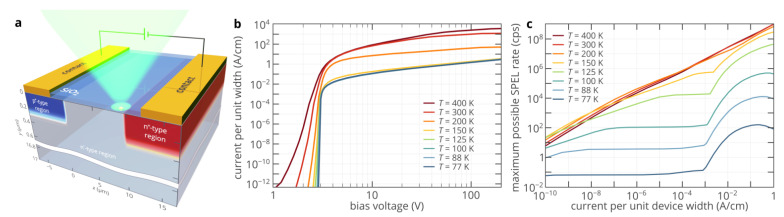
(**a**) Schematic of the 4H-SiC SPED based on a color center in the lateral p-i-n diode. (**b**) Simulated current–voltage characteristics of the diode in (panel **a**). (**c**) Dependence of the maximum possible SPEL rate on the current through the device at different temperatures.

Figure 2a presents a schematic of the 4H-SiC SPED where the color center in the n^−^-type region is pumped by a forward-biased lateral 4H-SiC p^+^-n^−^-n^+^ diode. The geometry and spatial distribution of donors and acceptors are the same as in Refs. [14,27,28], where such a SPED was studied experimentally and theoretically, except that the maximum dopant concentration was reduced from 10^19^ to 10^18^ cm^−3^ for better convergence at low temperatures. This, however, does not significantly affect the free carrier densities in the n^+^-and p^+^-type injection regions at room and lower temperatures, as shown in Figure 1d,e. The donor compensation ratio in the n-type region and acceptor compensation ratio in the p-type region are assumed to be 1% [29]. The contacts to the n-type and p-type regions are assumed to be ideal ohmic contacts. Although the contact resistance can significantly affect the current–voltage characteristic, especially at low temperatures, it has been demonstrated that the dependence of SPEL rate on the current in diamond SPEDs is very robust to the contact resistance variation [30]. Therefore, this assumption does not limit the generality of our results.

Figure 2c shows the maximum possible SPEL rate *R*_max_ of the SPED as a function of the current through the device. For each temperature and current, *R*_max_ is found as the SPEL rate of the ideal color center, i.e., the color center with a lifetime of the excited state *τ*_e_ << 1 ns and 100% quantum efficiency, for its optimum position in the n^−^-region of the 4H-SiC diode structure. If two charge states of the color are involved in the SPEL process (see Equation (1)), which is the most natural assumption for most color centers [14], the actual maximum single photon emission rate of the chosen color center can be found as [13]:

(2) $$R_{\max}^{\mathrm{cc}}={\left[\frac{1}{R_{\max}}\left(1+\frac{\tau_{r}}{\tau_{\mathrm{nr}}}\right)+\tau_{r}\left(1+\frac{\tau_{s}}{\tau_{\mathrm{nr}}}\right)\right]}^{-1}.$$

For a color center with two charge states participating in the SPEL process,

(3) $$R_{\max}=\frac{1}{c_{n}n}+\frac{1}{c_{p}p},$$

where *c*_n_ and *c*_p_ are electron and hole capture rate constants by the color center, respectively, while *n* and *p* are the electron and hole densities in the vicinity of the color center. The maximum possible SPEL rate characterizes the efficiency of electrical excitation rather than the inherent properties of a particular color center. If three charge states are involved in the SPEL process, the above dependences are slightly more complicated but quantitatively not very different [31]. The capture rate constants *c*_n_ and *c*_p_ depend on which charge states are involved in the SPEL process. Since multiple charge states do not participate in the SPEL process, we start with a color center with neutral and positively charged states since this corresponds to the silicon antisite defect, which is the most studied color center in 4H-SiC under electrical excitation [10,13].

**Figure 3:**
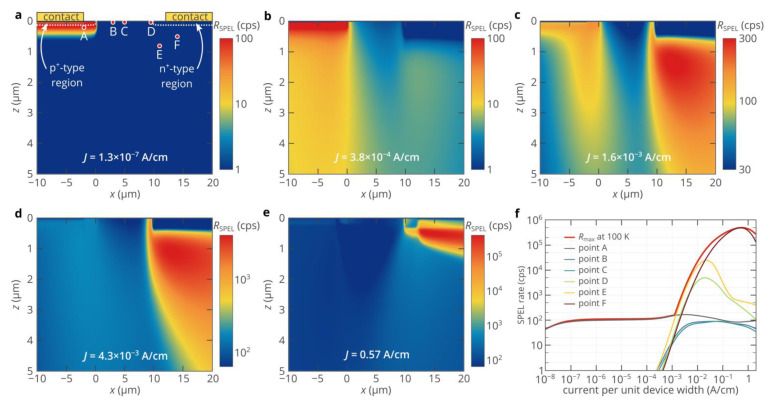
(**a**–**e**) Dependence of the SPEL rate of an ideal color center with two charge states involved in the SPEL process: positively charged and neutral—on its position in the 4H-SiC p^+^-n^−^-n^+^ diode at five different currents through the SPED. In panel a, the white dashed lines indicate the n^+^- and p^+^-regions. Points A, B, C, D, E, and F show the positions of the color center used in panel f. (**f**) Dependence of the SPEL rate of an ideal color center with two charge states involved in the SPEL process: positively charged and neutral—on the current through the SPED for different positions of the color center marked in panel a. Point A is chosen to be near the p^+^-n^−^ junction. Point B corresponds to the location of the color center in Refs. [14,27]. Point C is the middle between the n-type and p-type regions. Point D approximately corresponds to the optimal location of the color center at depths less than 50 nm. Point F corresponds to the optimal position of the color center. Point E is chosen to illustrate how the SPEL rate decreases if the color center is deviated from the optimal position.

At room temperature and high currents, *R*_max_ exceeds 10^9^ cps, which can give the possibility to emit more than 10^9^ photons/s if the lifetime of the color center is reduced below 1 ns. At 200 K, the maximum possible SPEL rate is of the same order of magnitude as at room temperature. However, at lower temperatures, the SPEL rate is significantly lower and rapidly decreases as temperature decreases (Figure 2c). For example, at 100 K, it is clearly seen that *R*_max_(*J*) increases as the current increases until it reaches 110 cps at *J* ≈ 10^−7^ A/cm and then saturates at this level. This saturation level is determined by the portion of ionized uncompensated acceptors in the p^+^-type region of the diode, as shown in the inset of Figure 1e. However, as *J* exceeds 10^−3^ A/cm, *R*_max_ suddenly starts to increase until it reaches its maximum of 5 × 10^5^ cps at *J* = 0.6 A/cm and then decreases. Such a dependence of *R*_max_ on *J* is in sharp contrast to that at 200, 300, or 400 K (Figure 2c). Moreover, while max(*R*_max_(*J*)) of the order of 100 cps can be expected from Figure 1e, the actual maximum of 5 × 10^5^ cps is more than three orders of magnitude higher than what can be expected from the temperature dependence of the hole density in the p^+^-region shown in Figure 1e. Qualitatively, the same dependence of the maximum possible SPEL rate on current is observed for all temperatures below 150 K (Figure 2c). To understand it and find how to achieve a SPEL rate of 5 × 10^5^ cps at 100 K, we plot and analyze the 2D spatial distributions of the maximum SPEL rate at different currents (Figure 3a–e).

**Figure 4:**
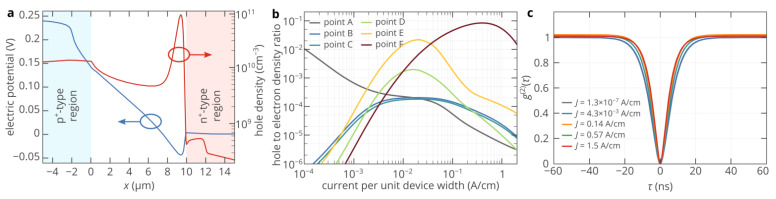
(**a**) Spatial distributions of the electrostatic potential and hole density along the *x*-axis at a depth of 130 nm at *V* = 3.3 V. (**b**) Ratio of the hole density to the free electron density at six different points inside the 4H-SiC SPED marked in Figure 3a. (**c**) Second-order autocorrelation function of the silicon antisite defect near stacking faults located at point F marked in Figure 3a, where the maximum SPEL rate can be achieved at 100 K for different currents through the device. The lifetimes of the excited and shelving states are assumed to be the same as at 300 K [14,27]. For all panels, the device temperature is equal to 100 K.

At a current per unit device width of 1.3 × 10^−7^ A/cm, the maximum SPEL rate is achieved if the color center is located in the p^+^-type region (Figure 3a). Since the electron density in the n+-region is many orders of magnitude higher than the hole density in the p^+^-region, electrons are injected into the n^−^- and p^+^-regions even at a low bias voltage, while the level of hole injection into the n^−^-region is very low. The maximum SPEL rate is about 110 cps, as predicted by Figure 1e, since the hole capture process appears to be a limiting factor. As the bias voltage increases, the band bending in the n^−^-region increases and holes start to be injected into the n^−^-region (Figure 3b). At the same time, the maximum hole density is still in the p^+^-region, as is expected from the standard theory of semiconductor devices [32,33], and therefore, the maximum SPEL rate is achieved if the color center is located inside or near the p^+^-region. The situation changes dramatically at bias voltages above ~3.05 V (Figure 3c–e). The maximum of the SPEL rate shifts toward the n^+^-region. Meanwhile, even at a current of 0.57 A/cm (Figure 3e), the SPEL rate in the p^+^-region is about 110 cps. Thus, the maximum SPEL rate is higher than the SPEL rate in the p^+^-region, where it logically should have been since the hole density is the limiting factor and the maximum hole density is expected to be in the p^+^-region of the p^+^-n^−^-n^+^ diode. Figure 3e shows that at high bias voltages, the SPEL rate right below the n^+^-region is more than three orders of magnitude higher than inside or near the p^+^-region, so is the hole density. The reason for this is the superinjection effect, which has been recently predicted to occur in homojunction diodes under certain conditions [34]. Figure 4a demonstrates that at bias voltages above 3 V, a potential well for holes arises near the n^−^-n^+^ junction due to the asymmetry in the hole conduction mechanisms in the p^+^- and n^−^-regions on one side and in the n^+^-region on the other side [34]: diffusion transport in the n^+^-region and drift transport in the p^+^- and n^−^-regions, which can be clearly seen by the slope of the electric potential in the corresponding regions (Figure 4a). Holes are accumulated in this potential well, while electrons almost do not see it since their density is much higher than the hole density at high bias voltages (Figure 4b). At very high bias voltages, the drift transport starts to dominate over the diffusion one even in the n^+^-region. Accordingly, as the asymmetry in the hole conduction mechanisms gradually disappears, so does the potential well near the n^+^-region [30]. Hence, the hole density and the SPEL rate decrease with the current (Figure 2c and Figure 3f).

It is noteworthy that the second-order autocorrelation function typically measured for all single-photon sources does not give any information about the brightness increase at high currents due to the superinjection effect, as seen in Figure 4c. The characteristic time of the *g*^(2)^ function almost does not depend on the pump current. The reason for this is that the SPEL rate is determined by the slowest process among the electron and hole capture processes, while the characteristic time of the *g*^(2)^ function is determined by the maximum between the lifetime of the excited state and the inverse time of the fastest carrier capture process min(1/*c*_p_*p*, 1/*c*_n_*n*) [35]. Since even under moderate bias, the electron density in the n^−^-region near the n^+^-region is orders of magnitude higher than the hole density (Figure 1e and Figure 4b), the electron capture process is much faster than the hole capture process. Moreover, since electrons are injected into the n^−^-region even at a low forward bias voltage, the electron density at the optimum position of the color center below the n^+^-region (point F in Figure 3a) almost does not depend on the pump current. Moreover, if the electron capture rate constant and doping level of the n^+^-region are high, 1/*c*_n_*n* is lower than the lifetime of the excited state, which makes the *g*^(2)^ function practically independent of the pump current.

**Figure 5:**
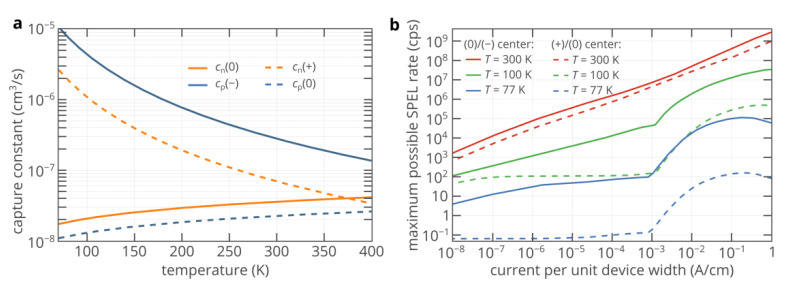
(**a**) Calculated temperature dependences of the electron and hole capture rate constants by the color center in the neutral, negative, and positive charge states. (**b**) Maximum possible SPEL rate as a function of the injection current at 77, 100, and 300 K for the (0)/(−) and (+)/(0) color centers at 100% quantum efficiency.

Above, we considered only the color center with positive and neutral charge states. At the same time, we mentioned that the electron and hole capture rate constants depend on the charge state of the color center. Since the electron and hole capture cross-section can vary from one color center to another, their accurate calculation and measurement could be challenging, especially if the electronic structure of the color center is not known [14,36,37]. Therefore, in order not to lose generality, we continue to estimate the capture rate constants by a charged color center within the cascade capture model [6,36], which is in fairly good agreement with the experiments [13,14,35], while we assume the capturecross-section by the neutral center to be 2 × 10^−15^ cm^2^ for both electrons and holes [38]. Figure 5a compares the temperature dependences of the electron and hole capture rate constants for different charge states of the color center. It is clearly seen that the hole capture rate constant by a negatively charged color center is orders of magnitude higher than the hole capture rate constant by a neutral center. Since the hole capture rate is the factor limiting the SPEL rate, at low temperature, the brightness of the SPED based on a color center that involves the neutral and negatively charged states in the SPEL process ((0)/(−) center) can be more than two orders of magnitude higher than that based on a center that involves the neutral and positively charged states ((+)/(0) center) (Figure 5b). For the (0)/(−) center, the SPEL rate can exceed 10^7^ photons/s, which is close to or even higher than the lifetime limit of many color centers in silicon carbide [2,39]. At the same time, the spectral properties at 100 K are much better than at 300 K.

If all three charge states—(1), (0), and (+1)—that can be involved in the SPEL process [13] are involved, our numerical simulations show that, at low temperatures, the SPEL rate is approximately equal to that of the (+)/(0) center if the color center electroluminesces from the (+1) charge states and is approximately equal to that of the (0)/(−) center if it electroluminesces from the (0) or (−1) charge states.

## 4. Conclusions

In summary, we have studied the temperature characteristics of single-photon emitting diodes (SPEDs) based on color centers in 4H-SiC. Although the densities of free charge carriers in the n-type and p-type injection regions of the SiC SPED rapidly decrease as temperature decreases and, therefore, the brightness of the 4H-SiC SPED at low temperatures is expected to be many orders of magnitude lower than at room temperature, we have demonstrated that the maximum SPEL rate can achieve the lifetime limit of the color center even at low temperatures, which is almost impossible using other wide-bandgap semiconductors, such as diamond. We have shown that thanks to the effect of the superinjection of holes in homojunction diodes, the SPEL rate can exceed 10^7^ photons/s even at 100 K, which gives the possibility to significantly improve the spectral properties and Debye–Waller factor of single-photon emission of color centers under electrical excitation while maintaining high brightness. It is important to note that such a high electroluminescence rate is not achieved in the whole volume of the 4H-SiC diode. The color center should be properly placed in the diode structure (Figure 3), which can be achieved using ion implantation [23] or laser writing [40] techniques. We emphasize that we report SPEL rates of the unoptimized structures. By optimizing the SPED, it should be possible to further increase the maximum SPEL rate at low temperatures.

## Data Availability

The data that supports the findings of this study is available from the corresponding author upon reasonable request.

## Supplementary Materials

The following are available online at https://www.mdpi.com/article/10.3390/nano11123177/s1, Table S1: Material parameters with temperature dependencies used in the numerical simulations.
