# Supplementary material for: Bright Silicon Carbide Single-Photon Emitting Diodes at Low Temperatures: Toward Quantum Photonics Applications
Source: Nanomaterials (Basel). 2021 Nov 24;11(12):3177. doi: 10.3390/nano11123177 (PMC8705877; doi:10.3390/nano11123177)
Supplement: Supplementary file 1 [file nanomaterials-11-03177-s001.zip › nanomaterials-1455328-supplementary.pdf]

nanomaterials

Supplementary Materials

Bright Silicon Carbide Single-Photon Emitting Diodes at Low Temperatures: Toward Quantum Photonics Applications

Igor A. Khramtsov and Dmitry Yu. Fedyanin *

Laboratory of Nanooptics and Plasmonics, Center for Photonics and 2D Materials, Moscow Institute of Physics and Technology, Dolgoprudny 141700, Russia; khramtsov@phystech.edu

* Correspondence: dmitry.fedyanin@phystech.edu

Table S1. Material parameters with temperature dependencies used in the numerical simulations.

Parameter	Model	Comments
Energy bandgap	$E_{\text{gap}}(T) = E_{\text{gap}}(0) - \frac{\alpha T^2}{\beta + T}$, where $E_{\text{gap}}(0)$ = 3.26 eV, $\alpha = 6.5\times10^{-4}$ eV/K, β = 1300 K	[1]
Electron SRH lifetime	$\tau^{\text{n}}_{\text{SRH}}(T) = \frac{\tau^{\text{n}}_{\text{SRH}}(300)\times\left(\frac{T}{300}\right)^{1.5}}{1+\frac{N}{N_{\text{ref}}}}$, where $\tau^{\text{n}}_{\text{SRH}}(300)$ = 340 ns, $N_{\text{ref}} = 10^{17}$ cm^{-3}	[2–4]
Hole SRH lifetime	$\tau^{\text{p}}_{\text{SRH}}(T) = \frac{\tau^{\text{p}}_{\text{SRH}}(300)\times\left(\frac{T}{300}\right)^{-0.5}}{1+\frac{N}{N_{\text{ref}}}}$, where $\tau^{\text{p}}_{\text{SRH}}(300)$ = 100 ns, $N_{\text{ref}} = 10^{17}$ cm^{-3}	[2–4]
Electron surface recombination velocity	$S_{\text{n}}(T) = const = 10^5$ cm/s	[5]
Hole surface recombination velocity	$S_{\text{p}}(T) = const = 10^5$ cm/s	[5]
Electron saturation velocity	Si-like dependence $\upsilon^{\text{n}}_{\text{sat}}(T) = \frac{\upsilon^{\text{n}}_{\text{sat}}(0)}{1+0.8\exp\left(\frac{T}{600}\right)}$, where $\upsilon^{\text{n}}_{\text{sat}}(0) = 5.1\times10^7$ cm/s, which gives $\upsilon^{\text{n}}_{\text{sat}}(300) = 2.2\times10^7$ cm/s	[6,7]
Hole saturation velocity	Si-like dependence $\upsilon^{\text{p}}_{\text{sat}}(T) = \frac{\upsilon^{\text{p}}_{\text{sat}}(0)}{1+0.8\exp\left(\frac{T}{600}\right)}$, where $\upsilon^{\text{p}}_{\text{sat}}(0) = 3.5\times10^7$ cm/s, which gives $\upsilon^{\text{p}}_{\text{sat}}(300) = 1.5\times10^7$ cm/s	[1,7]

Electron low-field mobility in the p⁺-type region	$\mu_n(T)=\mu_n(300)\times\left(\frac{T}{300}\right)^{-0.52}$, where $\mu_n(300)$ = 420 cm²/Vs	[8–10]
Hole low-field mobility in the p⁺-type region	$\mu_p(T)=\mu_p(300)\times\left(\frac{T}{300}\right)^{-0.93}$, where $\mu_p(300)$ = 170 cm²/Vs	[8–10]
Electron low-field mobility in the i-type region	$\mu_n(T)=\mu_n(300)\times\left(\frac{T}{300}\right)^{-1.59}$, where $\mu_n(300)$ = 700 cm²/Vs	[8–10]
Hole low-field mobility in the i-type region	$\mu_p(T)=\mu_p(300)\times\left(\frac{T}{300}\right)^{-1.59}$, where $\mu_p(300)$ = 220 cm²/Vs	[8–10]
Electron low-field mobility in the n⁺-type region	$\mu_n(T)=\mu_n(300)\times\left(\frac{T}{300}\right)^{-0.52}$, where $\mu_n(300)$ = 420 cm²/Vs	[8–10]
Hole low-field mobility in the n⁺-type region	$\mu_p(T)=\mu_p(300)\times\left(\frac{T}{300}\right)^{-0.93}$, where $\mu_p(300)$ = 170 cm²/Vs	[8–10]
Donor activation energy	$E_d(T)$ = *const* = 0.053 eV (hex site), 0.1 eV (cubic site)	[11]
Acceptor activation energy	$E_a(T)$ = *const* = 0.2 eV	[12]
Electron DOS mass in the conduction band	$m_e(T)$ = *const* = 0.77m_0, where m_0 is the electron rest mass.	[13]
Hole DOS mass in the valence band	$m_h(T)$ = *const* = 0.91m_0, where m_0 is the electron rest mass.	[14]

References

1. Levinshtein, M.E.; Rumyantsev, S.L.; Shur, M.S. *Properties of Advanced Semiconductor Materials: GaN, AIN, InN, BN, SiC, SiGe*.John Wiley & Sons:Hoboken, NJ, USA, **2001**; 98, 106–117.
2. Ščajev, P., Subačius, L., Jarašiūnas, K., Kato, A. Recombination and diffusion processes in electronic grade 4H silicon carbide. *Lithuanian Journal of Physics*, **2019**, *59*, 26–34.
3. Liaugaudas, G., Dargis, D., Kwasnicki, P., Arvinte, R., Zielinski, M., Jarašiūnas, K. Determination of carrier lifetime and diffusion length in Al-doped 4H–SiC epilayers by time-resolved optical techniques. *Journal of Physics D: Applied Physics*, **2014**, *48(2)*, 025103.
4. Li, X., Luo, Y., Fursin, L., Zhao, J. H., Pan, M., Alexandrov, P., Weiner, M. On the temperature coefficient of 4H-SiC BJT current gain. *Solid-State Electronics*, **2003**, *47(2)*, 233–239.
5. Kohama, K., Mori, Y., Kato, M., Ichimura, M. Evaluation of Temperature Dependence of Surface Recombination Velocities for n-type 4H-SiC, Extended Abstracts of the 2015 International Conference on Solid State Devices and Materials, Sapporo, Japan, 27–30 September 2015.
6. Khan, I. A., Cooper, J.A. Measurement of high-field electron transport in silicon carbide. *IEEE Transactions on Electron Devices*, **2000**, *47(2)*, 269–273.
7. Selberherr, S. *Analysis and simulation of semiconductor devices*. Springer-Verlag: New York, USA, **1984**, pp. 96.
8. Pernot, J., Zawadzki, W., Contreras, S., Robert, J.L., Neyret, E., Di Cioccio, L. Electrical transport in n-type 4H silicon carbide. *Journal of Applied Physics*, **2001**, *90(4)*, 1869–1878.
9. Erginsoy, C. Neutral impurity scattering in semiconductors. *Physical Review*, **1950**, *79*(*6*), 1013.
10. Sze, S.M., Ng, K.K. *Physics of Semiconductor Devices*, John Wiley & Sons: Hoboken, NJ, USA, **2006**, 28–50.

11. Evwaraye, A.O., Smith, S.R., & Mitchel, W.C. Shallow and deep levels in n-type 4H-SiC. *Journal of Applied Physics*, **1996**, *79(10)*, 7726–7730.
12. Ivanov, I.G., Henry, A., Janzén, E. Ionization energies of phosphorus and nitrogen donors and aluminum acceptors in 4 H silicon carbide from the donor-acceptor pair emission. *Physical Review B*, **2005**, *71(24)*, 241201.
13. Son, N.T., Chen, W.M., Kordina, O., Konstantinov, A.O., Monemar, B., Janzén, E., Meyer, B.K. Electron effective masses in 4H SiC. *Applied physics letters*, **1995**, *66(9)*, 1074–1076.
14. Son, N.T., Hai, P.N., Chen, W.M., Hallin, C., Monemar, B., Janzén, E. Hole effective masses in 4H SiC determined by optically detected cyclotron resonance. *In Materials Science Forum*, **2000**, *338*, 563–566.
